# A Global Transcriptional Switch between the Attack and Growth Forms of *Bdellovibrio bacteriovorus*

**DOI:** 10.1371/journal.pone.0061850

**Published:** 2013-04-16

**Authors:** Iris Karunker, Or Rotem, Mally Dori-Bachash, Edouard Jurkevitch, Rotem Sorek

**Affiliations:** 1 Department of Molecular Genetics, Weizmann Institute of Science, Rehovot, Israel; 2 Department of Plant Pathology and Microbiology, Faculty of Agriculture, Food and Environment, The Hebrew University of Jerusalem, Rehovot, Israel; University of Kentucky College of Medicine, United States of America

## Abstract

*Bdellovibrio bacteriovorus* is an obligate predator of bacteria ubiquitously found in the environment. Its life cycle is composed of two essential phases: a free-living, non-replicative, fast swimming attack phase (AP) wherein the predator searches for prey; and a non-motile, actively dividing growth phase (GP) in which it consumes the prey. The molecular regulatory mechanisms governing the switch between AP and GP are largely unknown. We used RNA-seq to generate a single-base-resolution map of the *Bdellovibrio* transcriptome in AP and GP, revealing a specific "AP" transcriptional program, which is largely mutually exclusive of the GP program. Based on the expression map, most genes in the *Bdellovibrio* genome are classified as "AP only" or "GP only". We experimentally generated a genome-wide map of 140 AP promoters, controlling the majority of AP-specific genes. This revealed a common sigma-like DNA binding site highly similar to the *E. coli* flagellar genes regulator sigma28 (FliA). Further analyses suggest that FliA has evolved to become a global AP regulator in *Bdellovibrio*. Our results also reveal a non-coding RNA that is massively expressed in AP. This ncRNA contains a c-di-GMP riboswitch. We suggest it functions as an intracellular reservoir for c-di-GMP, playing a role in the rapid switch from AP to GP.

## Introduction

*Bdellovibrio bacteriovorus* is an obligate predator of gram negative bacteria ubiquitously found in bulk soil, in the rhizosphere of plants, and in freshwater bodies such as rivers and water treatment facilities [1]. This organism can prey on a wide range of bacteria, including many pathogens of humans and plants, and has thus been suggested as an effective "living antibiotics" [2]. *B. bacteriovorus* displays a dimorphic life cycle, with two main phenotypes: Attack Phase (AP) and Growth Phase (GP). AP cells are small, fast-swimming, non-replicating "hungry" cells that search for prey, while GP cells are non-motile replicative cells that actively consume the prey [3]. *B. bacteriovorus* grows and replicates within the periplasm of the prey cells, using their cytoplasmic content as its source of energy and nutrients [4]. Prior to growth, the prey cell is killed and remodelled into a spherical bdelloplast. Growth within the prey cell takes place as an aseptate and polynucleoid filament with cell division controlled by the availability of soluble host components [5]. The GP cycle of *B. bacteriovorus* lasts about 4 hours, with the prey being killed within 30 minutes, and bdelloplast exhaustion and division of elongated *B. bacteriovorus* cells occurring about 3 hours after the initial attachment to the prey [6]. The cycle is completed by a subsequent rupturing of the bdelloplast envelope and a release of daughter AP cells.

Expression of *Bdellovibrio* genes during the switch from AP to GP phases was studied by Lambert *et al* using oligonucleotide microarrays [7]. This study reported 479 genes up regulated 30 minutes after the shift from AP to GP (early GP genes) and 230 genes that were downregulated (AP genes). In agreement with previous studies that were based on proteomics [8] the AP genes functions largely included motility, chemotaxis, adhesion and outer membrane genes, which are required for detection, attachment, and penetration into the prey cell [9], [10], [11]. The GP genes include those associated with growth and division such as ribosomal proteins and RNA polymerase components, as well as various transport and peptidoglycan metabolizing genes presumably involved in processing and internalization of prey materials [7]. Despite the recent advancement in understanding the genetic program differentiating between AP and GP gene expression [12], [13], the molecular regulatory cascade leading to the dramatic switch between the AP and GP phenotypes of *Bdellovibrio* is still largely unknown.

Transcriptome-wide cDNA sequencing (RNA-seq) is a modern method that is gradually replacing microarrays for bacterial transcriptome research [14]. This method allows more accurate quantification of RNA levels, and provides a complete coverage of expressed regions to a single nucleotide resolution [15]. It therefore allows a direct interrogation of the transcriptional landscape of a genome [14]. As opposed to microarrays, RNA-seq allows the detection of previously unannotated genes such as non-coding RNA regulators (ncRNAs). Indeed, RNA-seq studies in bacterial and archaeal organisms have revealed a plethora of functional ncRNAs and antisense RNAs, including the recently discovered long antisense "excludon" structures in *Listeria* [16], [17]. Specific RNA-seq protocols also allow genome-wide mapping of transcription start sites (TSSs), enabling single-base resolution mapping of promoter sequences for all expressed genes [17], [18], [19]. To date, no regulatory ncRNA has been reported in *B. bacteriovorus*, and its promoters are largely unmapped.

Here we investigated the organization of gene expression during the attack and growth phases in synchronized cultures of *B. bacteriovorus* using RNA-seq, mapping expressed genes as well as their transcription start sites (TSSs). Our data revealed a sharp dichotomy in gene expression between the two phases with, in the attack phase, exceptionally high levels of expression of a non-coding RNA containing a cyclic-di GMP riboswitch, and the expansion of gene expression control by a secondary sigma factor.

## Materials and Methods

### Bacterial strains and growth

*Bdellovibrio bacteriovorus* HD100 was grown in HEPES buffer (25 mM HEPES, 2 mM CaCl_2_. 2H_2_O, 3 mM MgCl_2_. 6H_2_O, pH 7.8), at 30°C and 250 rpm in two-membered cultures with *E. coli* MG1655 (ATCC #700926) as prey. Fresh attack phase (AP) cells were obtained from overnight cultures by inoculating 100 mL HEPES buffer with 2×10^9^ colony forming units (cfu) per mL of *E. coli* prey MG1655 and about 10^7^ mL^−1^ predatory cells.

### Synchronization of predatory cultures

Synchronization was obtained as previously described [7], [8]. Prey grown over night in NB at 37°C was diluted 1∶100 and cultivated under the same conditions for about 2.5 h, to an optical density (OD_570_) of 0.6 to 0.8. The cells were then pelleted at 4°C for 10 min at 4500 g, washed three times in HEPES buffer and resuspended in HEPES to a final volume of about 15 mL at OD_570_ ∼10. The suspension was then stored at 4°C overnight. A starter predatory culture was established by adding 5 mL of the prey suspension to 45 mL HEPES inoculated with 250 to 600 µL from a fresh *B. bacteriovorus* culture and grown for about 18 to 20 h, to yield 8×10^8^ mL^−1^ attack phase predators. This culture was then centrifuged for 10 min and 12,000 g at 25°C and resuspended in 2 mL HEPES, concentrating predatory bacteria by 25-fold to 2×10^10^ mL^−1^. The synchronized cultures were established by mixing 1.2 mL of the latter culture with 12 mL of prey suspension and 16.8 mL HEPES, resulting with ∼4∶1 excess of predators over prey and to a swift and unified infection of prey. Predation cultures were grown as above. Microscopic observations were taken throughout the procedure to verify culture synchrony.

### Sample collection

10 mL samples were retrieved at 60 and 180 min after mixing predator and prey, centrifuged at 1800 g at 4°C for 10 min, washed twice with cold HEPES, and finally resuspended in 1 mL cold HEPES. Bdelloplasts were purified from remaining attack phase *Bdellovibrio* cells by layering the suspension onto the surface of 30 mL of a Percoll (Sigma, Rehovot, Israel) solution in a 70 mL ultracentrifuge tube. The solution was prepared by mixing five parts of Percoll stock solution with four parts of 0.25 M sucrose (Barel). Centrifugation was performed at 50,000 g for 30 min at 4°C in a Sorvall® Centricon T-1170 with an A-641 rotor (Asheville, NC. USA). The upper band of purified bdelloplasts (∼ 4 mL) was washed with 5 volumes of 0.85% NaCl, centrifuged at 1800 g at 4°C for 10 min, the lower band being un-attacked prey cells. Percoll was removed from the fractions by dilution with five volumes of 0.85% NaCl and centrifugation at 20,000 g for 10 min 4°C and stored as a pellet at −80°C.

Attack phase cells were obtained from a 10 mL starter culture filtered through a 0.45 µm filter, to eliminate remaining prey cells. Cells were pelleted by centrifugation at 12000 g at 4°C for 10 min.

### RNA isolation

Pellets were resuspended in 5 mL TRI reagent (Sigma), homogenized 40 sec with a pipette and incubated 5 min at room temperature (RT). 0.5 mL of 1-bromo-3-chloropropane was added, manually mixed for 15 sec, incubated at RT for 10 min and centrifuged at 1500 g at 4°C for 30 min. The upper, transparent phase was then transferred to a clean tube, 2.5 mL isopropanol added and manually mixed, incubated at RT for about 10 min, then centrifuged at 1500 g at 4°C for 30 min. The RNA pellet was washed with 5 mL of a 75% ethanol solution prepared with DEPC-treated water, gently mixed, and centrifuged again at 1500 g at 4°C for 10 min. The ethanol was gently removed, the pellet was dried and resuspended in 200 µL nuclease-free TE (Sigma). The RNA samples were treated with TurboDNase (Ambion). rRNA depletion was performed using MicrobExpress (Ambion). RNA purity and concentration were determined using a NanoDrop spectrophotometer and Agilent Bioanalyzer.

### RNA sequencing

For the whole transcriptome sequencing, cDNA libraries were prepared by the Illumina mRNA-seq protocol according to the manufacturer's protocol without the polyA isolation stage. Sequencing was performed on an Illumina GAIIx sequencing machine. TAP(+) and TAP(-) 5'-end cDNA libraries for TSS mapping were prepared as previously described [17].

### Read mapping and expression calling

Genome sequences of *B. bacteriovorus* (Genbank: NC_005363) and *E. coli* (Genbank: NC_000913) were downloaded from NCBI. Sequencing reads were mapped to the genomes as previously described [17], [20]. The number of overlapping mapped reads was counted for every ORF in each sample of whole-transcriptome sequencing (AP and GP). For each ORF we calculated the number of reads per kilo-base of gene model per million mapped reads (RPKM) as previously described by Mortazavi *et al*. [21]; this procedure normalizes expression for gene sizes and for samples having different numbers of initial reads. Genes having RPKM above a threshold of 50 were defined as expressed in a condition. Differentially expressed genes were called by performing χ2 test on the RPKM values and applying Bonferroni correction for multiple-testing as previously described [22]. Genes with adjusted p-value smaller or equal to 0.05 were determined to be differentially expressed.

RT-PCR verification of mutually exclusive expression. Fresh AP *Bdellovibrio bacteriovorus* HD100 were inoculated onto an *Escherichia coli* ML35 culture (109 mL-1) to a final 5∶1 ratio. 25 minutes post inoculation predation culture was filtered through a 0.45 um nitrocellulose Microsart filter (sartorius) and the unfiltered bdelloplasts were collected and resuspended in HEPES buffer. 2 mL samples were taken from the AP culture and predation culture prior inoculation and after 0.5, 1 and 3 h, centrifuged and resuspended in RNAlater (Ambion). Total RNA was purified by TRI reagent (MRC, Inc.) and DNA contaminations were digested by TURBO DNA-freeTM kit (Ambion). Equal amounts of RNA were reverse transcribed with ImProm-IITM (Promega). Specific primers designed for representative genes were used to amplify cDNA from each time point (Table S3).

### Determination of TSS

Transcription start sites were determined based on relative enrichment of 5' end sequences in the TAP(+) sample as compared to the TAP(-) sample, as described in [17]. For this, 6.8 million and 7.5 million 5' end reads were mapped to the *Bdellovibrio* genome and analyzed for the TAP(+) and TAP(-) samples, respectively. Positions were called as TSSs if they were (i) supported by at least 5 reads; (ii) showed at least 3-fold enrichment in the TAP(+) sample; and (iii) positioned upstream of a gene. TSSs were further curated manually to remove redundancies and to control for problematic gene annotations.

### Genome wide detection of sRNAs

Reads were counted in each intergenic region, and the potential for differential expression between the AP and GP conditions was assessed by χ2 test with correction for multiple testing as described above for protein coding genes. Intergenic regions showing >4 fold increased expression in the AP phase were further interrogated manually. Predicted long 5'UTR sequences, manifested by expression connected to the downstream gene, were discarded. The beginning and strand orientation of predicted sRNA was determined by the TSS data; the end of the sRNA was estimated by the coverage of the whole-transcript RNA-seq data. The RPKM for each sRNA was adjusted based on the sRNA coordinates, and differential expression was re-calculated. Predicted sRNAs were searched for homology against the entire protein collection (nr) using blastX with a minimal e-value of 10^−4^. Putative ORFs with homologs in other organisms were removed from the list. The remaining predicted sRNAs were searched against RFAM (http://rfam.sanger.ac.uk/) for known RNA family sequence motifs.

## Results

### Mutual exclusiveness in attack- and growth-phase transcriptional programs

To attain the transcriptional landscape of *B. bacteriovorus* under different growth phases, RNA was extracted either from (i) attack-phase *B. bacteriovorus*; (ii) *B. bacteriovorus* synchronically growing within *E. coli* 1 hour after infection; and (iii) *B. bacteriovorus* synchronically growing within *E. coli* 3 hours after infection. RNA was sequenced using the Illumina high throughput sequencing technology, yielding short reads of 36bp (Table 1). Mapping of reads to the genomes of *B. bacteriovorus* and *E. coli* enabled separation between RNA derived from the predator and that derived from the prey; this mapping revealed that 1 hour post infection, 99.6% of the RNA is still that of the prey, whereas 3 hours post infection, RNA of the predator increased 13-folds (Table 1). The scarceness of *B. bacteriovorus* sequence reads in the 1 hour sample prevented global-scale transcriptome analysis for that time point, and therefore the analyses presented henceforth refer to the comparison between attack-phase (AP) and growth phase 3 hours post infection (GP).

**Table 1:** RNA-seq data used in this study.

Sample	# Reads	% mapped to *Bdellovibrio*	# reads mapped to *Bdellovibrio* protein coding genes
**Attack phase**	34,857,665	99.18%	3,309,352
**Growth phase 1hr**	34,062,256	0.40%	82,169
**Growth phase 3hr**	32,632,726	5.16%	987,791

**Figure 1:**
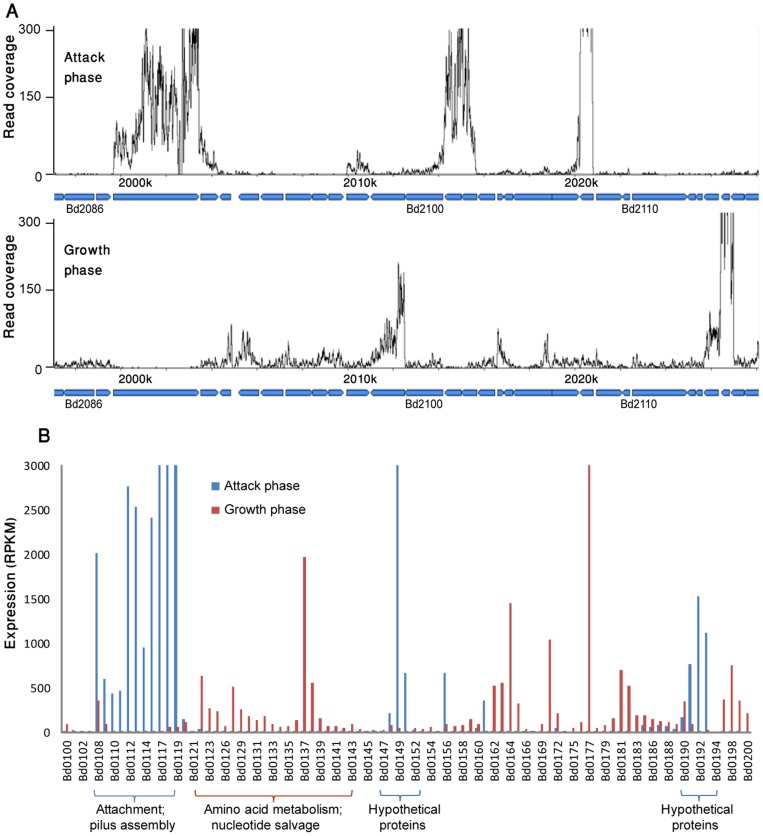
Mutual exclusive expression of attack phase and growth phase genes. (A) Expression of a ∼30 kb genomic region between genes *bd2086* and *bd2118*. X-axis, position on the genome; Y-axis, a per-base transcript coverage map. (B) Expression of a window of 100 genes between *bd0100*-*bd0200*. Y-axis, normalized expression per gene calculated as reads per kb per 1 million reads in the library (RPKM)[21].

Sixty-five percent of the genes in the *B. bacteriovorus* genome showed noticeable expression under at least one of the studied growth phases (Table S1). Strikingly, the expressed genes showed an almost mutually exclusive expression pattern with respect to the phase: 67% of the expressed genes (1557 genes) were active in the GP but were silent in the AP, whereas an additional 15% of the genes (353 genes) were exclusive to the AP phase but showed no expression in GP. Moreover, even in the minority 18% of genes that were transcribed in both phases, many of these showed a differential expression of at least 5-fold, being dominant either in the AP (114 genes) or in the GP (66 genes). Only 250 genes were expressed in both phases with less than 5-fold difference between conditions. Thus, the *B. bacteriovorus* demonstrates an extreme case of transcriptional switch between AP and GP, and these two growth phases are dominated by transcriptional programs which are almost completely mutually exclusive (Fig 1).

**Figure 2:**
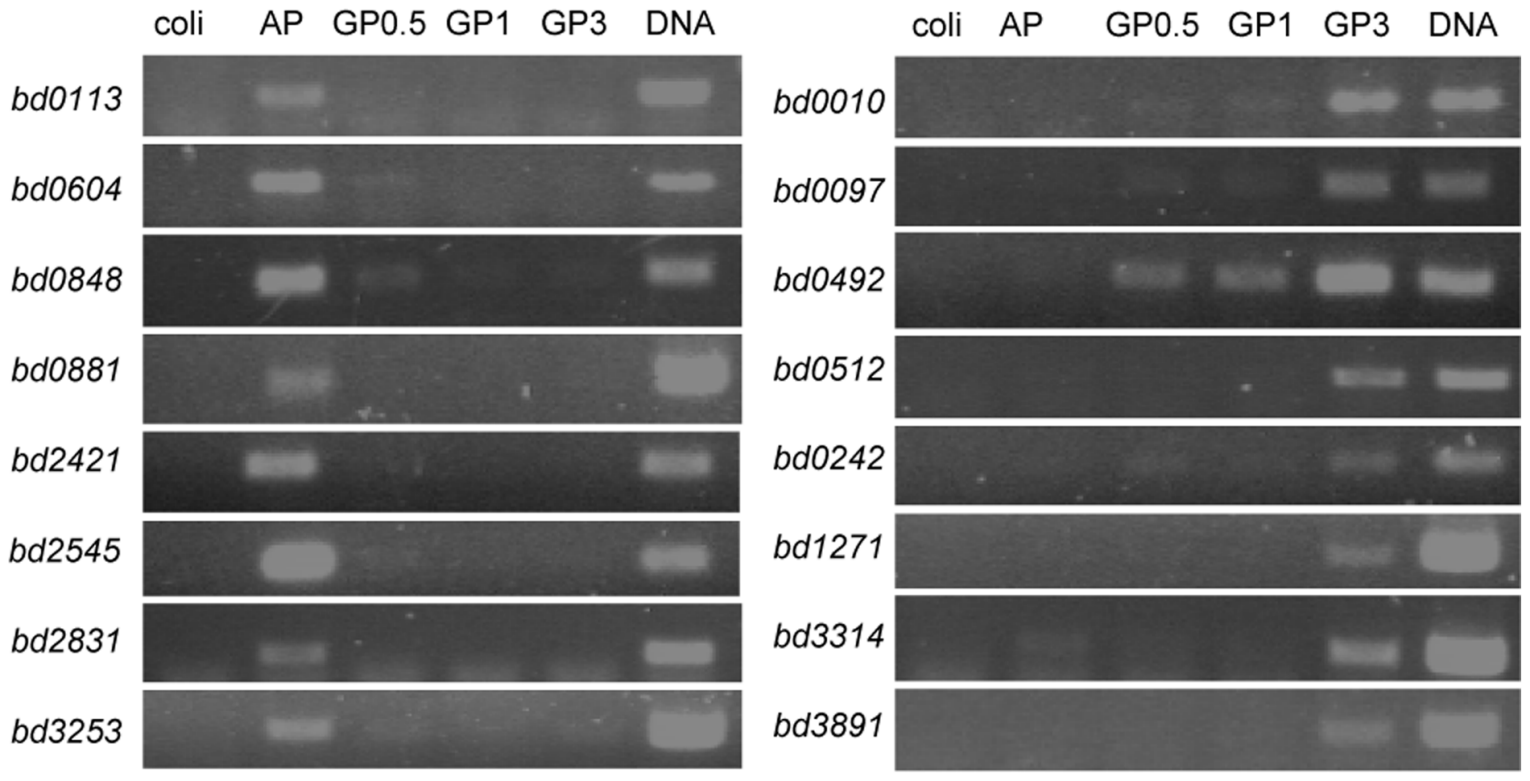
RT-PCR verification of mutually exclusive expression. Total RNA retrieved from AP or GP *B. bacteriovorus* HD100 during a synchronous predation of *E. coli* ML35 (0.5, 1 and 3 hrs post inoculation) was subjected to RT-PCR. Sixteen representative genes predicted by RNA-seq analysis to be AP-specific (left) or GP-specific (right) were amplified. Coli, control genomic DNA of *E. coli* ML35; AP, cDNA from AP cells; GP0.5, cDNA of GP cells 0.5 hr post inoculation; GP1, cDNA of GP cells 1 hr post inoculation; GP3, cDNA of GP cells 3 hrs post inoculation. DNA, *B. bacteriovorus* genomic DNA.

To independently verify the mutually exclusive expression patterns observed in the RNA-seq data, we performed RT-PCR on 8 genes determined as AP-specific and 8 genes marked as GP-specific in the RNA-seq analysis. In all cases, mutual exclusiveness was clearly observed (Fig 2).

Of the 353 AP-specific genes that we identified, 219 (62%) were also found to be overrepresented in AP conditions in the Lambert et al study [7]. In general, most genes identified by us but not in the earlier study had lower expression values, probably pointing to the higher sensitivity of the RNA-seq method as compared to microarrays [15], [23]. The functions of AP-specific genes largely followed what was previously described, with 59% of these genes coding for uncharacterized "hypothetical" proteins, and the remaining mostly involved in motility, chemotaxis, and cell-surface composition (Table S1). The overlap between our GP-specific set (1557 genes) and the Lambert GP-upregulated genes (479 genes) was smaller, most probably due to the different time points examined in the two studies (30 minutes and 180 minutes post infection in the Lambert and the current study, respectively). The functions encoded by the GP-specific genes were largely those associated with cell growth, including ribosome biogenesis, cell division, DNA polymerase and chromosome partitioning proteins, and energy metabolism (Table S1). Since the functions of AP and GP genes have been discussed in breadth in other studies [7], [8], we here focus mainly on analyzing the global transcriptome structure and the derived regulatory insights.

**Figure 3:**
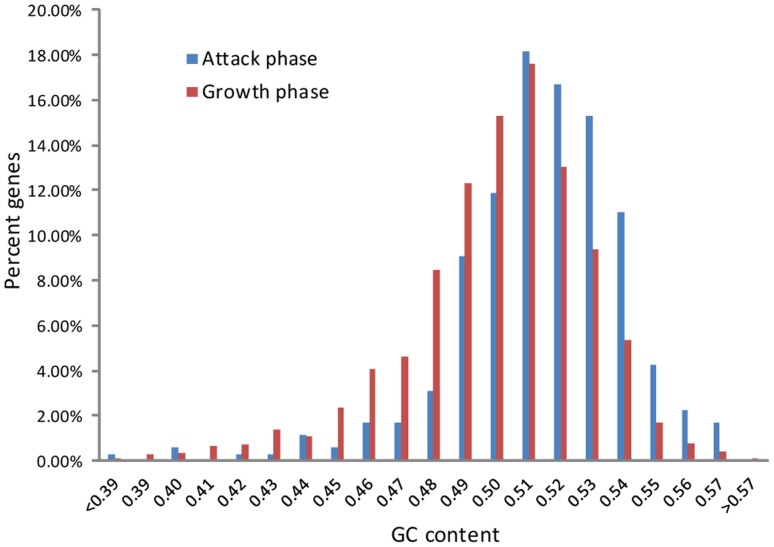
GC-content distribution of attack-phase and growth-phase genes.

Visual examination of the transcriptional landscape across the genome revealed that AP- and GP-specific genes are largely clustered on the genome, forming islands of program-specific genes (Fig 1). The *B. bacteriovorus* genome therefore appears to be composed of a mosaic of program-specific patches, with each patch containing multiple AP- or GP-specific genes. The two groups of genes differ slightly (but significantly) by their GC content (*p* = 1.9×10^−15^, T-test; Fig 3). These results may suggest that different selective pressures are acting on AP and GP genes, and the traces of these different pressures are manifested in the GC content; alternatively, these results may point to different ancient origins of AP and GP genes (see Discussion).

### Regulation of attack phase genes

To further explore the regulation of the AP transcriptional program switch we performed genome-wide transcription start sites (TSSs) mapping using massive sequencing of RNA 5' ends (Methods). We have previously demonstrated that such TSS maps, generated using a sequencing method that relies on selective Tobacco Acid Pyrophosphatase (TAP) treatment to differentiate between primary transcripts and processed RNAs, can map active promoters in a genome wide manner to a single-base resolution [17], [18]. Since the massive fragmentation of *E. coli* prey RNA in the GP phase impedes high quality 5' end sequencing, we focused on promoters active in the AP phase only. We were able to map 140 AP-specific promoters, which overall control the majority (232 genes) of AP-specific genes in the *B. bacteriovorus* genome through regulation of mono- and poly-cistronic mRNAs (Table S2).

**Figure 4:**
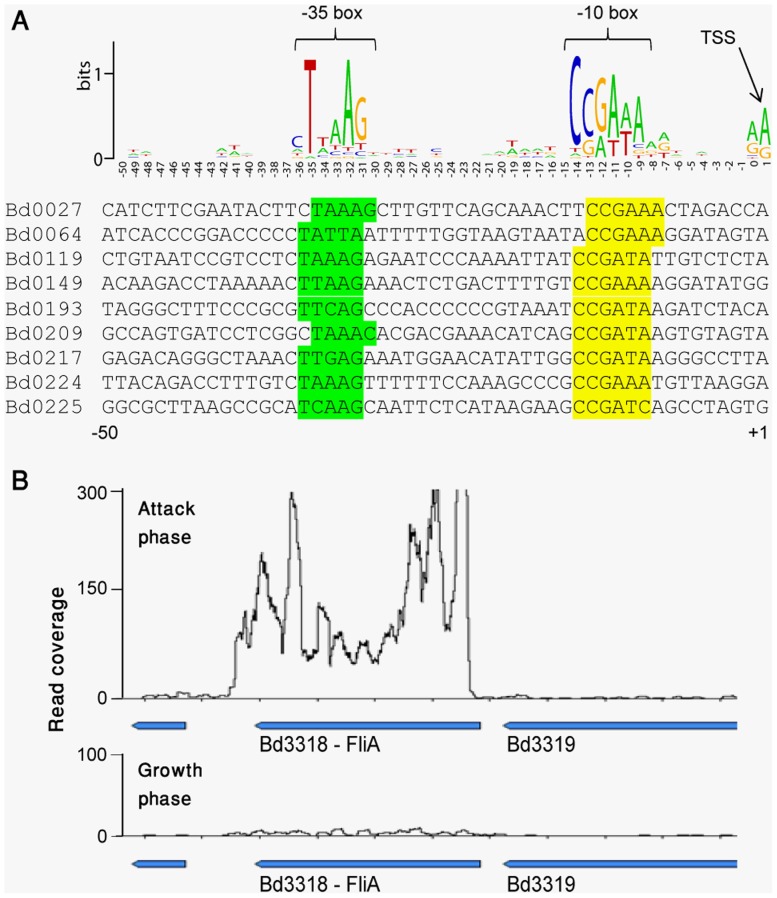
FliA binding site in AP-specific promoters. (A) Sequences enriched in AP-specific promoters. AP-specific transcription start sites (TSSs) were determined using massive sequencing of RNA 5' ends. Fifty bases upstream of each determined TSSs were taken and analyzed for enriched position-specific sequence signals using the online tool weblogo [40]. The presented logo represents consensus sequence from 140 AP-specific promoters; shown below is a representative set of such promoter sequences. (B) Expression of gene *bd3318*, coding for FliA, in attack (top) and growth (bottom) phases. X-axis, position on the genome; Y-axis, a per-base transcript coverage map based on RNA-seq.

Searching for sequence motifs in the AP promoter sequences retrieved a strong sigma-like consensus of TTAAG-N16-CCGATA, which appeared as a −35 and −10 box in 92/140 (66%) of the AP-specific promoters (Fig 4A). In *E. coli* and other gram negative bacteria, this sequence was documented as the binding site of the FliA (sigma28) transcriptional regulator [24], [25], [26]. Indeed, the FliA-like gene itself (*bd3318*) shows a strong AP-specific expression in *B. bacteriovorus*, further suggesting that FliA is a major regulator of the AP-specific transcriptional program (Fig 4B).

In many bacteria including *E. coli* [27], *Salmonella* [28], *Legionella pneumophila* [29], and *Vibrio cholera* [30], FliA is known to regulate flagella biosynthesis by activating flagella-specific genes. However, in *B. bacteriovorus* FliA appears to regulate two-thirds of the AP-specific program, which includes (in addition to flagella) many non-flagellar genes such as cell-surface anchors, proteases, chemotaxis proteins and many uncharacterized proteins (Table S2). These results therefore suggest that in *B. bacteriovorus* FliA has evolved from a flagella regulator to become a global regulator of the AP-program.

We were not able to identify a strong sequence motif in the 33% remaining promoters that did not present a FliA binding site. It is possible that these promoters are regulated by a mixture of more specific regulators; indeed, we found the FliA binding site in the promoters of two predicted transcriptional regulators (*bd0931* and *bd1891*) that have AP-specific expression, and the sigma factor sigE (*bd0881*) is also expressed in an AP-specific manner (Table S1). These additional regulators might control the expression of the non-FliA regulated AP-specific genes.

### Attack phase-specific small RNAs

The availability of a genome-wide expression and TSS maps now allows searching for non-coding RNAs in the *B. bacteriovorus* genome. Small non-coding RNAs (sRNAs) are gaining high attention in recent years as regulators of important processes in bacteria such as virulence, quorum sensing, and stress responses [16]. We were able to find 8 AP-specific novel sRNAs transcribed from intergenic regions, and mapped their exact start sites and strand directionality using the TSS data (Table 2). Most of these sRNAs have strong potential for RNA secondary structures (Fig S1). Each of these sRNAs was at least 16-fold over-represented in the AP condition, with two extreme cases reaching 5000-fold over representation. Three of the sRNAs contained a strong FliA motif in their promoters (Table 2). These sRNAs are therefore suspected as involved in regulating AP-specific functions in *B. bacteriovorus*.

**Table 2:** AP-specific small RNAs.

	Start	End	Str.	Size	Norm. Expression AP (RPKM)	Norm. Expression GP (RPKM)	Fold increase in AP	FliA promoter?
**APsRNA1**	499,375	499,581	+	207	50,451	3,181	16	
**APsRNA2**	535,188	535,321	+	134	8,869	204	43	
**APsRNA3**	561,854	561,981	+	128	14,726	77	191	yes
**APsRNA4**	562,984	563,081	+	98	3,500	30	116	
**APsRNA5**	605,441	605,617	−	176	388,842	73	5295	
**merRNA**	1,073,741	1,074,185	−	445	2,038,479	407	5006	
**APsRNA6**	1,338,787	1,339,441	+	655	6,809	147	46	yes
**APsRNA7**	3,416,607	3,416,881	+	275	32,816	70	469	yes

**Figure 5:**
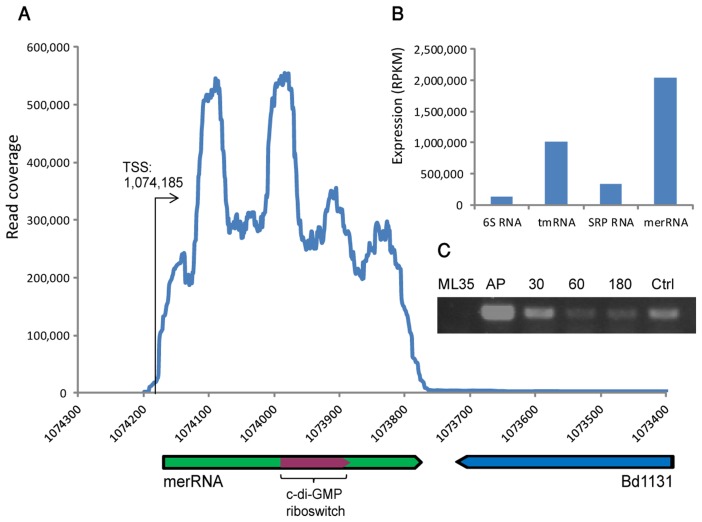
An AP-specific, massively expressed, riboswitch-containing sRNA. (A) Genomic organization and expression of merRNA. X-axis, position on the *B. bacteriovorus* genome; Y-axis, coverage of RNA-seq data. (B) Normalized expression of merRNA and other structural RNAs in attack phase. Expression is presented in RPKM [21] values, which are normalized for gene size and for the amount of reads in the library. (C) RT-PCR verification of merRNA expression being dominant in attack phase (AP) and reduced with infection of *E. coli* (30,60, and 180 mins after infection). Ctrl, *B. bacteriovorus* genomic DNA; ML35, genomic DNA of *E. coli* ML35.

Strikingly, one of the identified sRNAs was massively expressed in the attack phase (Fig 5). This sRNA, located in genomic coordinates 1,073,741−1,074,185 on the reverse strand (sized 445 nt), is covered by over 3 million sequencing reads. To appreciate this extraordinarily high expression, we note that this single sRNA is covered by a number of reads that is roughly equal to the number of all reads covering the entire set of protein coding genes in the *B. bacteriovorus* genome in AP conditions. Such unusually massive transcription is expected from structural RNAs such as rRNA or tmRNA (transfer-messenger RNA), rather than from regulatory RNAs; this sRNA was 6-fold more expressed than the housekeeping SRP RNA, and 2-fold more expressed than the highly abundant tmRNA (Fig 5B). Interestingly this sRNA harbours a cyclic-di-GMP (c-di-GMP) riboswitch, a secondary metabolite that regulates motility and biofilm formation in many bacteria [31]. We denote this RNA merRNA (massively expressed riboswitch RNA).

The merRNA is conserved in another recently sequenced *Bdellovibrio* strain, *B. bacteriovorus* JSS (Jurkevitch and colleagues, unpublished), but we could not find evidence for conservation outside the *Bdellovibrio* lineage. We could not find a conserved open reading frame downstream to the riboswitch, implying that this riboswitch is not functioning in the classical way as a regulator of a protein-coding gene expression. The massive transcription of merRNA, typical of a structural RNA, also argues against a classical riboswitch function for this sRNA. We propose that merRNA is functioning as an intracellular storage for c-di-GMP, enabling rapid response to changing conditions (see Discussion).

### The Bdellovibrio transcriptome browser

In order to make the transcriptome map and the promoters straightforwardly accessible for the research community, we have generated an online web browser that presents the *B. bacteriovorus* AP and GP transcriptional maps. (http://www.weizmann.ac.il/molgen/Sorek/bdello_browser/viewer/index.php?meta=bdello_combined&jump=bdello,bdello_sRNAs#1 [user: deltapro ; password: vtech999]).

This interactive browser contains several layers of information, including annotation of genes, comparative transcriptional maps of AP/GP expression, TSSs and sRNAs. It allows navigation between desired genes, visually comparing their expression between the GP and AP conditions and relative to other genes, browsing TSSs and small RNAs, and retrieving the exact DNA positions of promoters and other desired elements.

## Discussion

Numerous studies have examined differential expression of genes in bacteria under different growth conditions both via microarrays and RNA-seq. Usually, the majority of genes in the studied organism are expressed to some extent (with as much as 99% of the genes showing expression in *Burkholderia cenocepacia* [22]), and a minority of genes are reported to change their expression patterns between the conditions. Our finding that the vast majority of genes in *B. bacteriovorus* are differentially expressed represents an extreme case of transcriptional program separation. Moreover, expression patterns such as the one observed for the AP form, where the vast majority of the genes is completely or almost completely silenced, has, to our knowledge, never been reported in bacteria before. Such a unique pattern is probably the outcome of tight regulation activating the right genes at the right time and preventing expression leakage. This extremely dichotomic transcriptional program mirrors the extremely dichotomic life style of *B. bacteriovorus*, with the dramatic changes in its growth forms reflected in dramatic changes in the underlying transcriptional program.

The set of AP-specific genes we identified largely overlaps the set reported previously when the transition between AP and GP (30 minutes post infection) was studied using microarrays [7]. However, the extreme transcriptional switch that we observed was not reported in that study. This probably reflects the tendency of microarrays to provide relative but not absolute measures of expression, and their lower dynamic range and sensitivity as compared to RNA-seq [15], [23]. As a result, annotating genes as completely silenced is challenging in microarray analyses. It is also probable that the transcriptional switch between AP and GP (3 hours), which we have studied, is more dramatic than the switch between AP and GP (30 minutes) that was studied by Lambert *et al* [7].

The largely segregated transcriptional programs (Fig 1A), along with the patchy distribution of AP gene clusters in the genome (Fig 1B) and the difference in GC content between AP and GP genes (Fig 3), is intriguing. It may lead to the speculation that in the ancient *B. bacteriovorus* ancestor AP-genes were originally separated from GP genes, possibly found on a megaplasmid, which merged into the genome during evolution through multiple recombination events. However, several lines of evidence contradict this scenario: first, the GC content of wobble positions is not significantly different between the two sets of genes (data not shown); second, it was predicted that 20% of the genes in *B. bacteriovorus* have been transferred from Gram negative bacteria outside the δ-proteobacteria lineage [32], but the genes predicted to be transferred are both AP and GP genes. Therefore, the observed differences in GC content between AP and GP genes may reflect a functional constraint, where AP genes are more enriched for amino acids coded by codons rich in G's and C's. Alternatively, increased GC content in AP genes may reflect higher tendency for RNA secondary structures, known to affect transcript stability [33].

Our results provide a promoter map for the majority of AP-specific genes. Two thirds of these promoters carry a clear FliA consensus sequence, strongly suggesting that FliA is the key regulator of most AP-specific genes. In *E. coli*, FliA is a late regulator of flagellar gene expression [24], and this role was also shown for FliA in many other bacteria [28], [29], [30]. Although flagella is an important feature of the AP *B. bacteriovorus*, it appears that FliA has adopted a more global role in the AP regulatory program, controlling not only flagella genes but also cell-surface proteins, proteases, and many other AP genes. Additional experiments, including FliA knockouts, are needed to further establish this point. Additional sigma factors probably control the remaining 33% of AP-specific genes, in agreement with the recent observation that deletion of the RpoE-like sigma factor in *Bdellovibrio* results in less efficient predation [13]. Indeed, our data show that the rpoE gene (*bd3314*) is also AP-specific (Table S1).

The identification of 8 AP-specific ncRNAs is the first report on ncRNAs in *Bdellovibrio* (Table 2). Additional, GP-expressed ncRNAs may also exist but the lack of reliable TSS data for GP RNA prevents determination of the premises of these ncRNAs and the strand from which they are expressed. In bacteria, small non coding RNAs (sRNAs) are known to regulate important processes such as quorum sensing, iron-metabolism and virulence [16]. Frequently, the regulatory function is exerted by base-pairing of the sRNA to the 5' end of target mRNAs, resulting in inhibition of translation or mRNA degradation [16]. It is therefore possible that the APsRNAs we detected (Table 2) regulate AP mRNAs in a similar manner. There was no significant homology for these APsRNAs in other genomes except for that of *B. bacteriovorus*, further suggesting that these sRNAs perform *Bdellovibrio* AP-specific regulatory functions.

One of the AP sRNAs we detected, which is expressed to extraordinary levels, contains a c-di-GMP riboswitch (Fig 5). In bacteria, the vast majority of riboswitches occur at the 5'UTR of protein coding genes, regulating these genes in *cis* either transcriptionally or translationally [34]. Some riboswitches were found to function also at the 3' end of genes as transcriptional terminators [35]. Other riboswitches have dual roles, functioning both as *cis*-regulators of gene expression and as *trans*-acting noncoding RNA regulators [36]. Riboswitches that have no role in *cis*-regulation of a protein coding gene and that function as part of a non-coding RNA were only rarely described. In one report, a riboswitch was shown to regulate gene expression from the opposite strand in an antisense-RNA mechanism [37]. However, the extreme expression of the riboswitch-containing merRNA suggests a structural role for this riboswitch rather than a *trans*-acting regulatory role.

Cyclic-di-GMP is a second molecular messenger controlling, in many proteobacteria, the transition between motile and biofilm lifestyles [31], or the transition from free living to pathogenic behaviour [38]. In *B. bacteriovorus* there are four active diguanylyl cyclase (DGC) genes that can produce c-di-GMP, and multiple other genes that can sense this molecule [12]. Three of the four DGC genes are AP-specific, with the remaining gene showing little-to-no expression in both AP and GP conditions, indicating a role for c-di-GMP signalling mainly in the attack phase (Table S1). Consistently, serial deletion of DGC genes in *B. bacteriovorus* abolished gliding motility (*dgcA*), impaired predation (*dgcB*), and changed AP cell morphology (*dgcC*) [12]. The mechanisms by which these *dgc* genes exert these different functions are currently unknown. Our finding of a ncRNA containing a c-di-GMP riboswitch adds another level of complexity to the intracellular c-di-GMP communication network.

The c-di-GMP riboswitch has a very high affinity to c-di-GMP, with a K_D_ of ∼1 nM [39]. Therefore, the presence of huge amounts of this riboswitch expressed in the cell as part as the merRNA are expected to result in a sponge-like action where significant quantities of cellular c-di-GMP will be absorbed into the riboswitch in the merRNA. With this in mind, one may speculate a role for merRNA in the AP-to-GP transition. According to this speculation, changes in the intracellular environment resulting from attachment to the prey, reflected by a change in a specific metabolite or the pH, will lead to structural changes or degradation of merRNA, thus resulting in massive release of c-di-GMP into the cell cytoplasm. In turn, this released c-di-GMP will regulate the rapid and extensive transformation of *B. bacteriovorus* from its attack, motile form to a sessile growth-phase form. This model, of course, is yet to be validated.

## Supporting Information

Figure S1 Potential secondary structures in AP sRNAs, as predicted by RNAfold.
(PDF)

Table S1 Gene expression in the attack phase (AP) and growth phase (GP3). Overlapping mapped reads were counted for every ORF in the AP and GP3 samples, and the RPKM was calculated for each ORF. RPKM>50 signifies phase-dependent expression. A χ2 test on the RPKM values with Bonferroni correction for multiple-testing was apply to call for differential expression, determined by an adjusted p-value <0.05 (green, in AP column; red, in GP3 column).
(XLSX)

Table S2 Mapped attack phase (AP) promoters. 140 AP-specific promoters controlling 232 AP-specific genes in mono- and poly-cistronic mRNAs.
(XLSX)

Table S3 Primers used for RT-PCR of specific gene.
(DOC)
