# Supplementary material for: A Global Transcriptional Switch between the Attack and Growth Forms of Bdellovibrio bacteriovorus
Source: PLoS One. 2013 Apr 16;8(4):e61850. doi: 10.1371/journal.pone.0061850 (PMC3627812; doi:10.1371/journal.pone.0061850)

Figure S1. Potential secondary structures in APsRNAs, as predicted by RNAfold

```
>APsRNA1
TTATAGTTTCAGACACATGGACGTGTTTGAAACGCGGGGAGGGTGAGAAAGAGATCGCGAACCATGGAAGGTCAAG
AGTCAAAAGTTTCTCGCAGGAAACAGGAGCAATAGGTCAGCAGAGATCACGGAGTGATCAACACGAGCCGAAGCTA
CACTCTCAGAATCGAGTGCAGGATGCACATGATTCTCCGACTCTAACTCCCCTTT
```

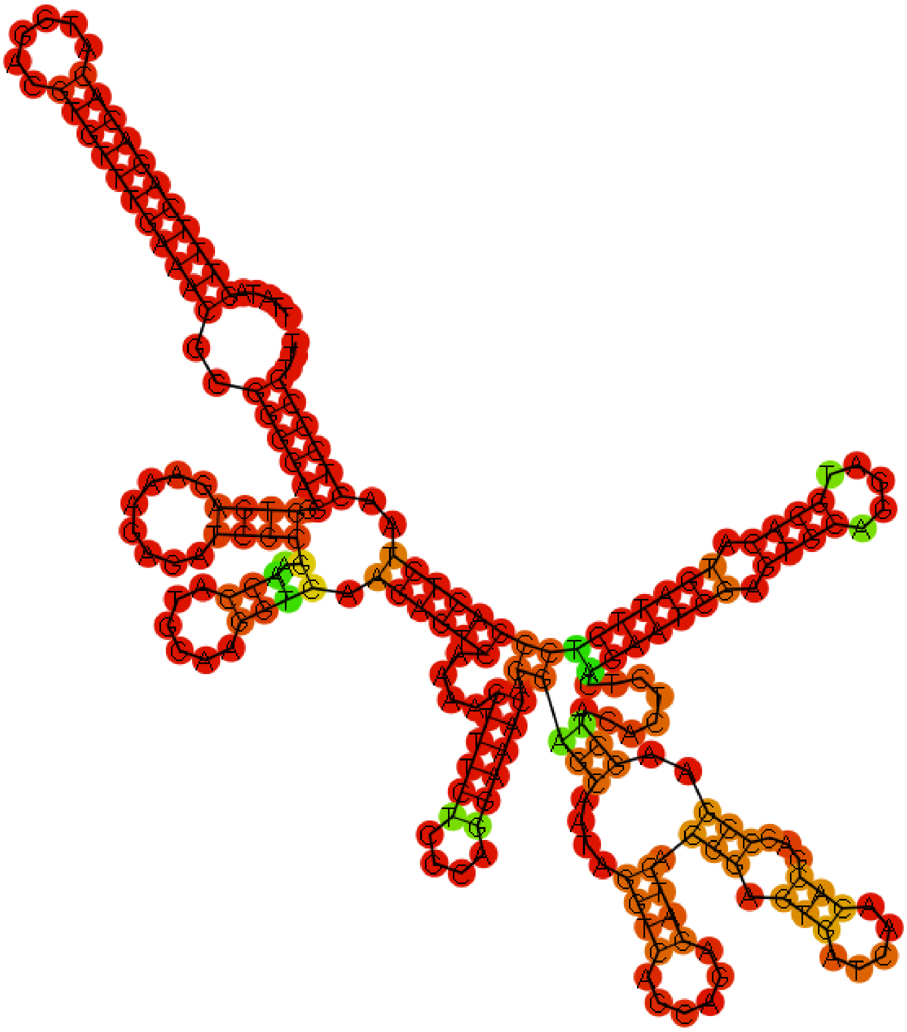

>APsRNA2
AAGCTTCTATTGGGGCTGGTAAGGAGGTGATGCTTATGGTTAAAAACGTAGACATAACCAAGGCGGAGGTGACGTC
CTCTTAGGACGCCTAGACCTTCGCCGAGGGTTCGAAAGAATCATAAACCTAAGAGCGA

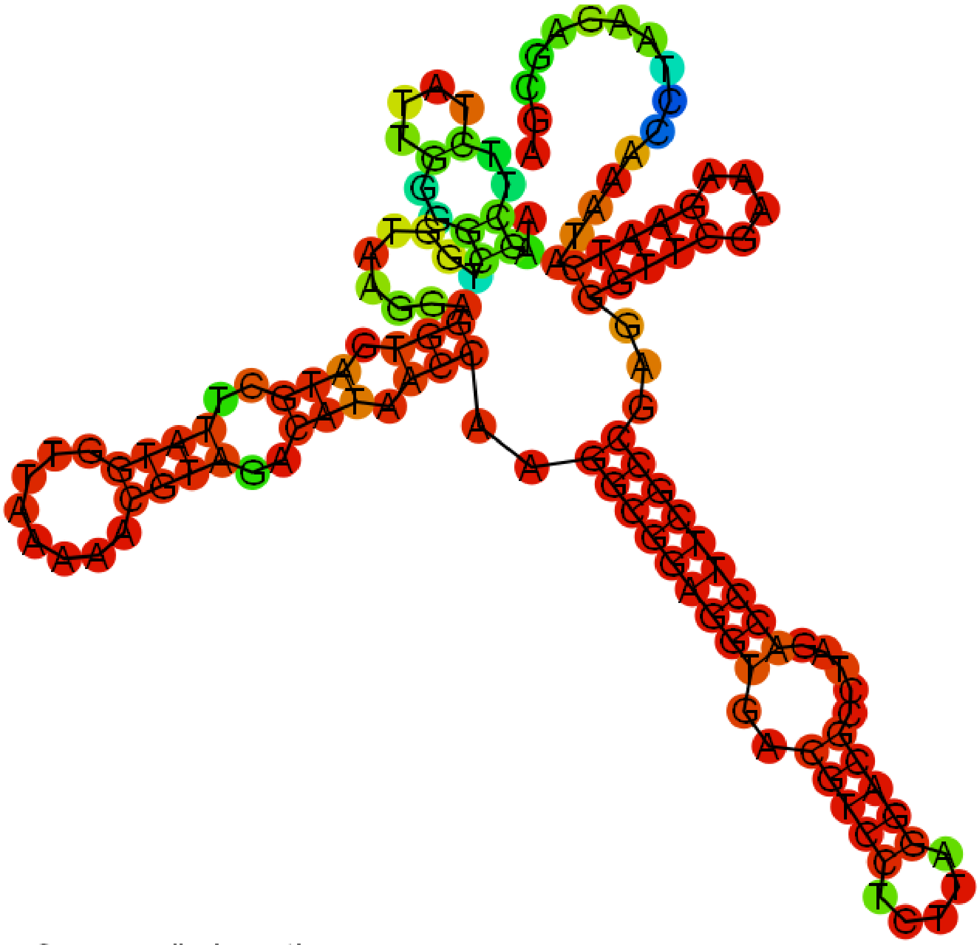

>APsRNA3
ACGTTGGGCAAGCTAAAAAGGATCTCCAGATCAAATCAGGATACCTCCTCTCGAAGCTCAAGCTCCTCTCAAGAAA
CGTCCAATCACCGTTTCTTAAGAGGGGCGGCTCCAACCTTAAATAAATTTTTTAA

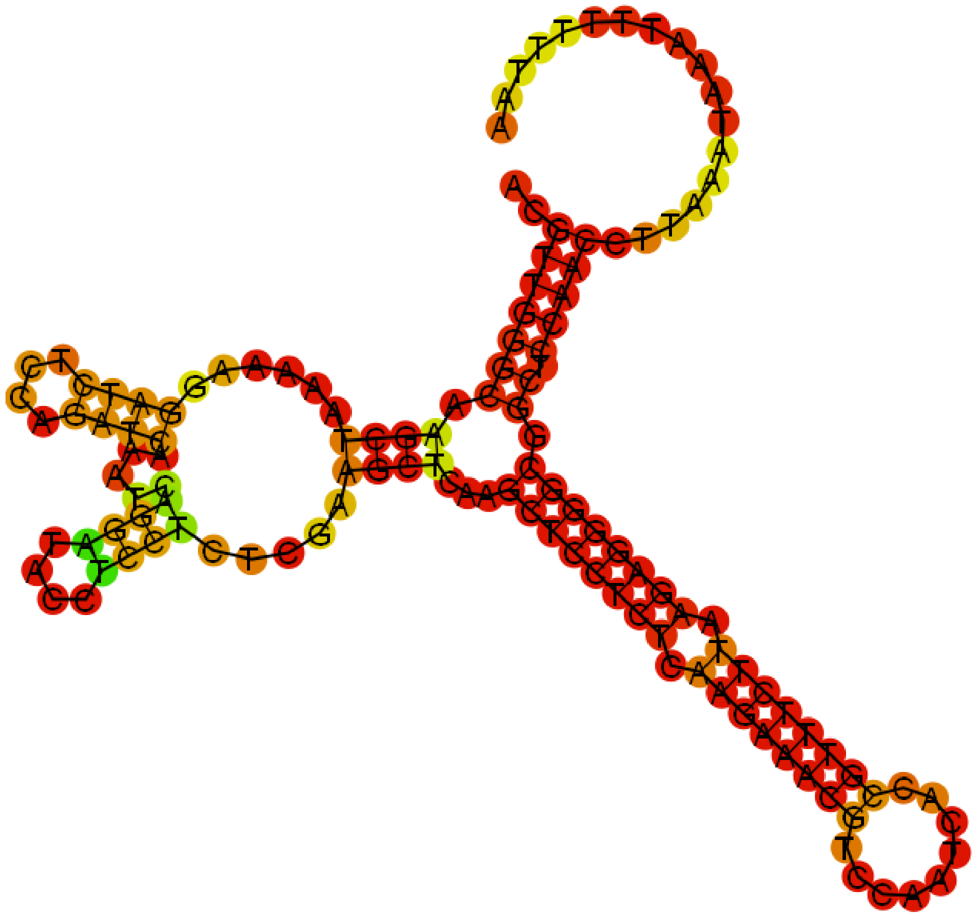

>APsRNA4
ATAATATCAGCGTAAAATATTTAAGATTCACATTCGCGATTACGGAATGTCGGGAGAAGCCTGTTCTGGGATAAGG
CTTCTCTCGGGAAGAGGGGTCGCCC

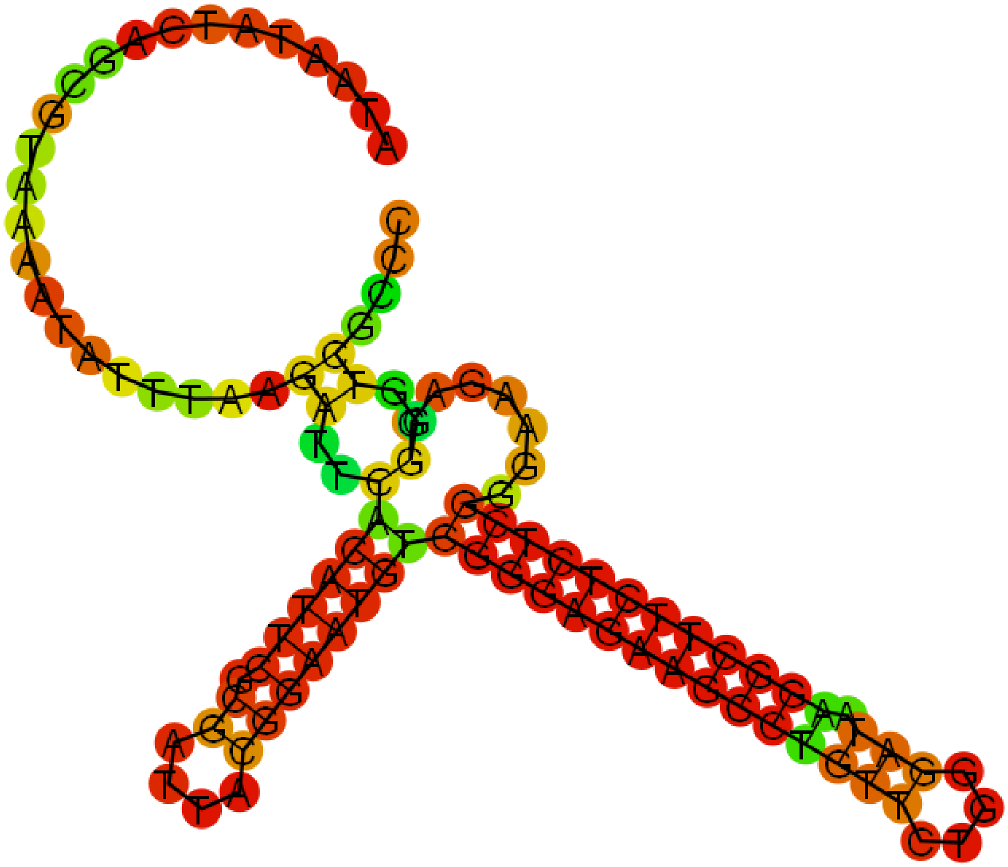

>APsRNA5
GAACCTCGGGTTAGGTTCTGCGAAAGGAGCCTTGAATGGCTAAAAAGCATGTCGTACGTAAGGAATATATCTGGAA
CCGGGAGATTCGTGAATTGGCTCTCCTGACAGAGACGGGTCCACCTTAAGTGACAAGATCAGATTTTTGATTGGTG
ATTAAATAAAAAAACCAGAGCGAATAAGGCGCCCTGGTTTT

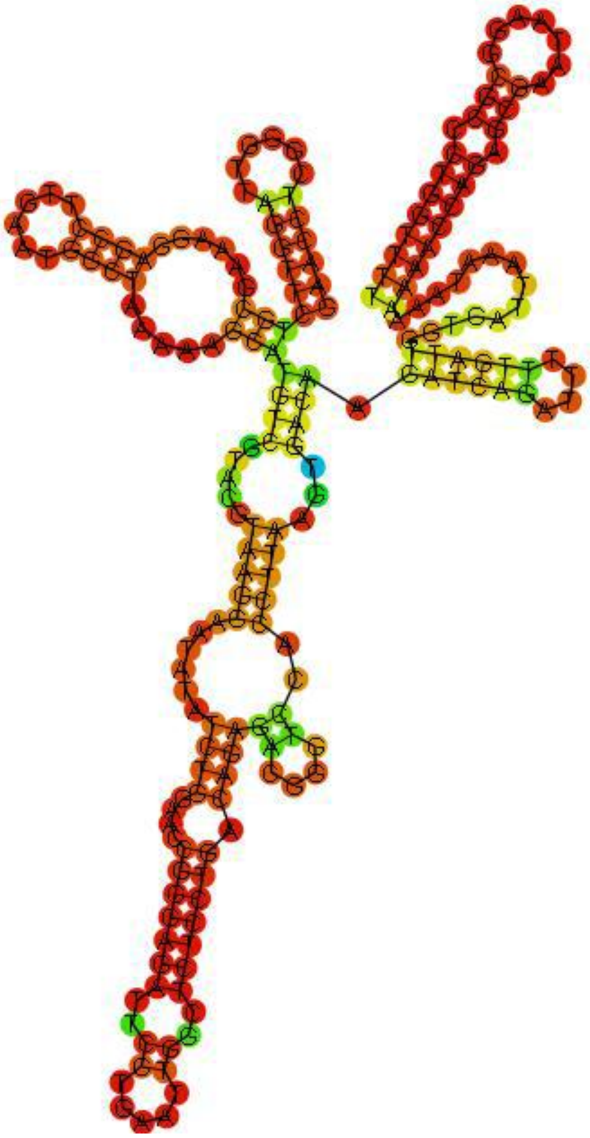

>merRNA
CAAGGCTCGGGAGACTTACCTCGGGTAAGTGAACGCTAGTAGGGCCAAGCGAAGGAGGACACGGATGAGAACACAT
GAACATCACGATGACGAACTGAAGTGAAGATGCGTCACCAGGATTACTACAAAGAGGTTGGGTTCATGCCTCTCTA
AAGAAGAATGGACTGGTTGGATCTGATGAATAACTCATAAGGAGGTTCCTAGGGTTATACTCGTTAGGATATAGAT
CCTTCCTTTGCCTCAAGGCAAACCGTCGGAAACGGCGGGACGCAAAGCTAAAGGGGTGTCGGTGCAAAGCCACGCC
AGCCAGCTGCCAAAGGTCATGAGTCACCTGAGGAATCAGAGGTGTGCATATGGAAGGAACAAATACATACAAGGAT
TGGTTTCATTCCCCTTAGCTAGCGAGTTATCACTGACGATTCCTCGCTAGCACTTTTTTTGGCGA

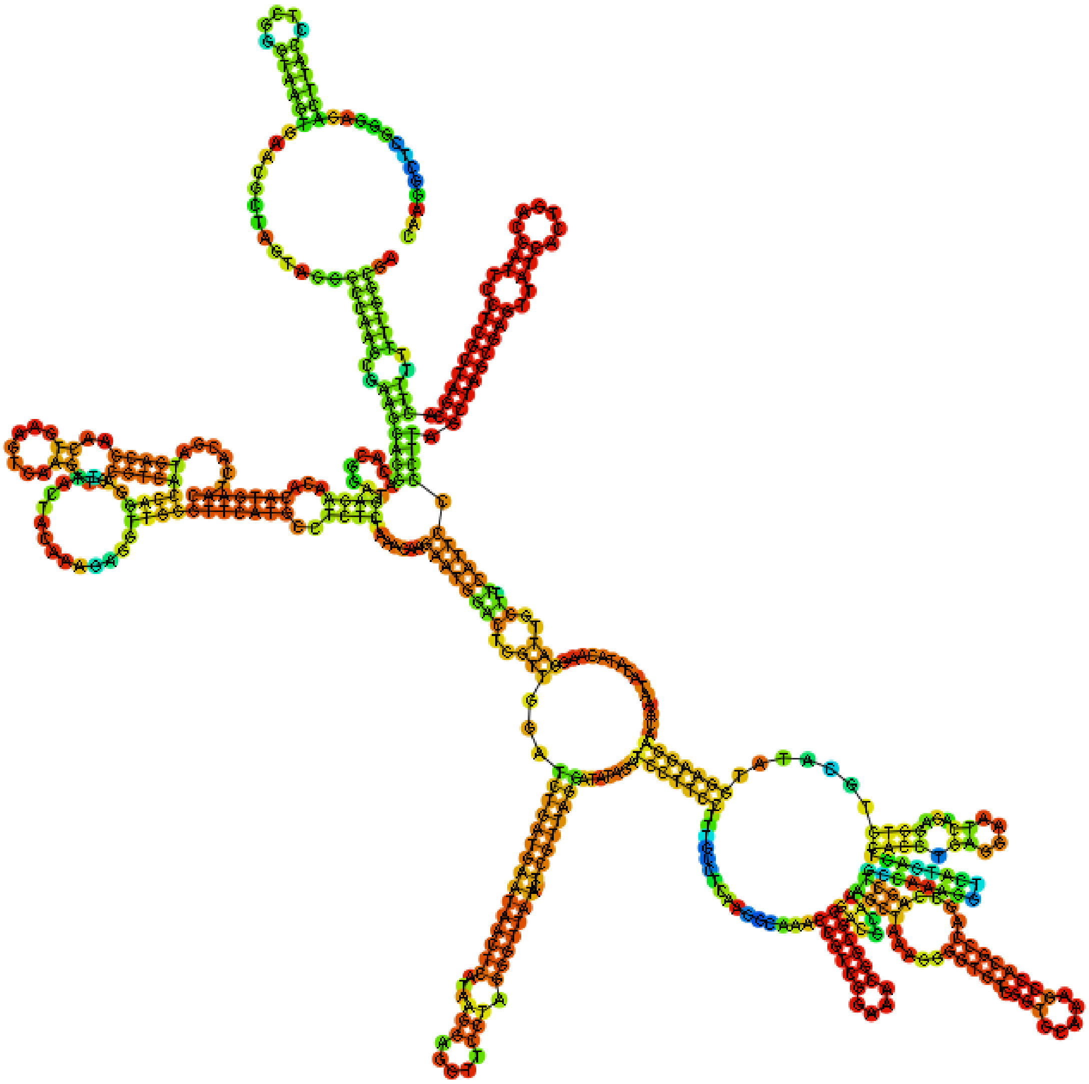

>APsRNA6
GAAATAACCAAAATAATTGAACGTAGGTGGTGGTTATGAATACGGAGCTTGGCGCATTTGCGCTCAGGCAGGTGCT
TGCCTCAACAGCAGTTCTTGGGCCGATCTTAATCCTGATCGGACTCTGCTACTTCCTCTTTAACCAAAGCAGAAGA
AGCGTGACCTTATACATAGGCACGGGGTTGATTCTGACCGTCATAGGAGTCACCTGTTTGTACTCCGAACTTCGAA
GCGAAGATTCGCGGGCTCACCATGAAGCAGCCATCGAACAGGTCGAAGTCATCGCGGAGGAAGCTGTTCGGAAGTA
GCTTCTGAATTGCTTGGTCCGTTTTGGCGCGGGGATTTGCTGGCGAACTTGGCCAGTCCCAGCGCTACGGGGGACG
CCGTCCGGGCTCAGTTGTCGTTCGGCCCGCCGGGCCGAATGCGCGCCATCCAGGCGCCGACAAAGCCCCCTTCGCG
CTGGCACTGTCCAAGCTCACCAGCAAACTTGCGTTGTCCCAATCAATTCAGAAACAACTTCCGAAAATCCCATCAC
AAAGACATCGGTTCCAAAGGGCATCGTCGTGCACCTGCGCAGTTAAGAACACATTGATAGTTATTAGCTATTTCCG
ACTGGAACAATTTGGATGGGGACTTGAGGGAGCCCGACGCAGCGGTC

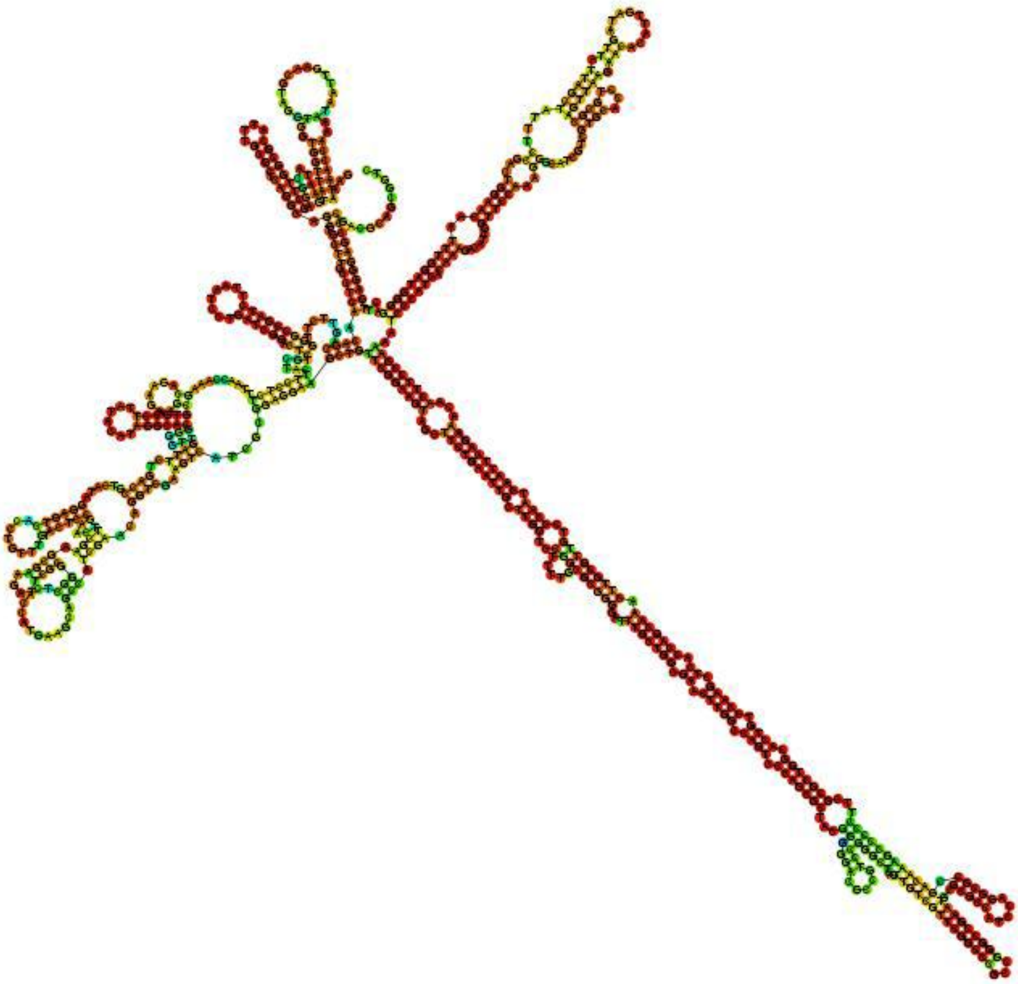

>APsRNA7
GTGTGACCATTAGGACTCAGTGTGGAGGCAAGTATGTTGAATAGAGGTAGTTTTAGTTTCGTAGCGATGCTGGTAA
TCAATCCCGTCGTGATACTGTTTTATCAAAACTGCTCGATGGCGCCGGTAAGCCACGCCAAAAACTCCACACCAAT
CCCTGTGATCACCCGCCAAGTGGCTTCCGAGTCACAGCCGGTGGTTGCCGATATCAAACCGGTCTGCCACGACGAA
AAGAAAGAATGCCCACGTAAGGAATAATATTCTTTACGTGCATATCT

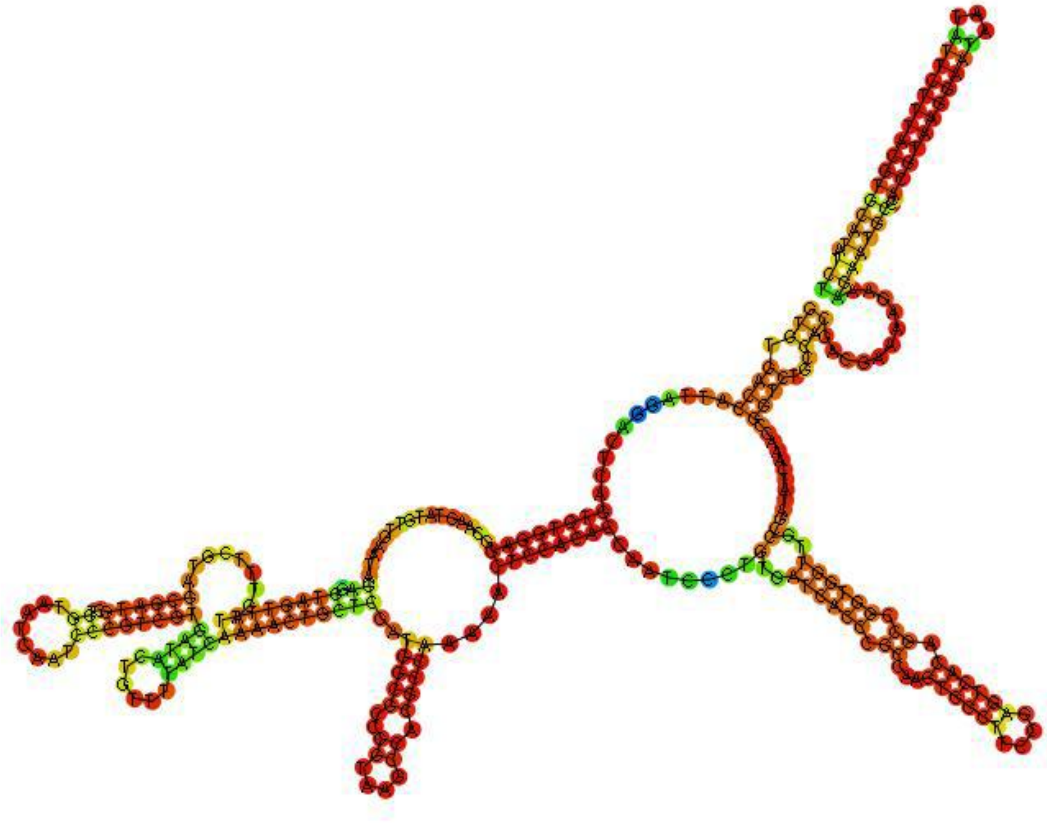

Supplement: Figure S1 — Potential secondary structures in AP sRNAs, as predicted by RNAfold. (PDF) [file pone.0061850.s001.pdf]
