# Supplementary material for: A Global Transcriptional Switch between the Attack and Growth Forms of Bdellovibrio bacteriovorus
Source: PLoS One. 2013 Apr 16;8(4):e61850. doi: 10.1371/journal.pone.0061850 (PMC3627812; doi:10.1371/journal.pone.0061850)
Supplement: Table S3 — Primers used for RT-PCR of specific gene. (DOC) [file pone.0061850.s004.doc]

**Table S3. Primers used for RT-PCR of specific genes**

| **Representative gene** | **Gene product*** | **Forward primer (5’>3’)** | **Reverse primer (5’>3’)** |
| --- | --- | --- | --- |
| *bd0113* | Flp pilus assembly protein CpaB | CGACGTTCCTGCTCTACA | TTCAACCATCGTGTCATAGA |
| *bd0604* | Flagellin | CAACAAACACCGCATCTAT | TCCTTTGAAGTTCTCAGAAAT |
| *bd0848* | Ribosome-associated protein Y | ACCACTCTGAATCCCTTGA | GGAGATCTCAACGCAGAAT |
| *bd0881* | RNA polymerase sigma factor RpoE | GACTTTGCGAAGTTCTATGA | CGGAACTTGTTTCGATGT |
| *bd2421* | Integral HD domain-containing protein | CATTCCAACTTGGGCTTA | GCTTCTGCTGATACTCATTTA |
| *bd2545* | Subtilisin-like serine protease | GTGACGCCGATTCTAAGA | GAAGAGGCTTTGAACTTGAT |
| *bd2831* | Methyl-accepting chemotaxis protein | GGAGAGACCTTCGATGAGTA | TCGAGGTTGAATTCAATCA |
| *bd3253* | Chemotaxis MotB protein | CTTTATGACGGCCTTGAT | CGAAGTTCTGGAAATTGTAT |
| *bd0010* | ATP synthase subunit C | TCGCTATGTTCGCATCTT | CACCTGCGAATACAGAGATA |
| *bd0097* | Chaperonin GroES | GCCCACTTCATGACAGAA | CTTTATCGCCAACTTTAACT |
| *bd0492* | 50S ribosomal protein L13 | TGGTCGTGTAGCAACTCAT | CTTCTTGTCCTGCCACTTA |
| *bd0512* | Recombinase A | TAGACAACAAAGCGAACTCA | GATATCCAAGCTCAATGCA |
| *bd0242* | RNA polymerase sigma factor RpoD | AAGAGCCTGTGAAGGTTCT | GATGGAGCGACAATTTCA |
| *bd1271* | Periplasmic protease | TGAAGGATACGAAACGAAT | TTCTTCCAGCTTGTCGAT |
| *bd3314* | RNA polymerase sigma factor RpoH | CTCCAAAGCGCTGACTT | CAGGGTCTTTGCTTTCAA |
| *bd3891* | NADH dehydrogenase I chain H | ATGGGAATGGGTAAAGATAT | ACCCAGACGATCCTGAAT |

* Gene products are presented as in the NCBI database
